# Tau or neurofilament light—Which is the more suitable biomarker for Huntington’s disease?

**DOI:** 10.1371/journal.pone.0172762

**Published:** 2017-02-27

**Authors:** Valter Niemelä, Anne-Marie Landtblom, Kaj Blennow, Jimmy Sundblom

**Affiliations:** 1 Department of Neuroscience, Neurology, Uppsala University, Uppsala, Sweden; 2 Clinical Neurochemistry Laboratory, Institute of Neuroscience and Physiology, The Sahlgrenska Academy at University of Gothenburg, Mölndal Campus, Mölndal, Sweden; 3 Department of Neuroscience, Neurosurgery, Uppsala University, Uppsala, Sweden; Centre de Recherche Jean-Pierre Aubert, FRANCE

## Abstract

**Introduction:**

Previous studies have suggested cerebrospinal fluid (CSF) levels of neurofilament light (NFL) and total tau are elevated in Huntington’s disease (HD) and may be used as markers of disease stage. Biomarkers are needed due to the slow disease progression and the limitations of clinical assessment. This study aims to validate the role of NFL and tau as biomarkers in HD.

**Methods:**

CSF was obtained from a cohort of HD patients and premanifest HD-mutation carriers. Unified Huntington’s Disease Rating Scale (UHDRS) testing was performed on all subjects at the time of sampling. NFL and tau concentrations were determined by ELISA. Spearman correlations were calculated with R version 3.2.3.

**Results:**

11 premanifest HD and 12 manifest HD subjects were enrolled. NFL and tau levels were correlated. NFL showed strong correlations with all items included in the clinical assessment (for example the total functional capacity (TFC) (r = - 0.70 p < 0.01) and total motor score (TMS) (r = 0.83p < 0.01). Tau showed slightly weaker correlations (eg. TMS (r = 0.67 p < 0.01); TFC (r = - 0.59 p < 0.01)). NFL was significantly correlated with 5-year probability of disease onset, whereas tau was not.

**Conclusion:**

This study strengthens the case for NFL as a useful biomarker of disease stage. NFL was strongly correlated to all evaluated items in the UHDRS assessment. Tau also has a potential for use as a biomarker but correlations to clinical tests are weaker in this study. We suggest that NFL and possibly tau be used in clinical drug trials as biomarkers of disease progression that are potentially influenced by future disease-modifying therapies.

## 1. Introduction

Huntington’s disease (HD) is an autosomal dominant inherited neurodegenerative disease caused by a CAG expansion of the HTT-gene. The triplet expansion gives rise to a cascade of downstream pathophysiological events that are still subject to intense research. The leading hypothesis is that mutant huntingtin (mtHTT) interacts with other proteins and alters their function, resulting in impaired axonal transport, disturbed energy metabolism, dysregulation of gene transcription, protein aggregation [1], and decreased neurotrophic support in the striatum. The last offers an explanation of why the striatum is severely affected [2]. Surprisingly, due to a process called RAN-translation, mtHTT is not the only disease-causing protein that is expressed from an expanded HTT-gene, and this new finding warrants further investigation [3].

The symptoms of HD include motor impairment and chorea, cognitive decline and a variety of psychiatric symptoms. Current treatment options are purely symptomatic, as there are no disease-modifying drugs. However, several promising clinical trials are underway [4].

One difficulty in performing HD trials is the lack of `wet`biomarkers for disease progression, and currently trials rely on clinical evaluation susceptible to symptom fluctuations and inter-rater variability. Some promising candidate biomarkers recently suggested remain to be validated [5]. In the search for biomarkers, cerebrospinal fluid (CSF) has an advantage over blood because of its proximity to the neurodegenerative process. Biomarkers are needed in clinical trials due to the slow disease progression and the limitations of clinical assessment [6].

Neurofilament triplet is a family of structural proteins of neurons especially found in their axons present in the white matter of the brain. Neurofilament light subunit (NFL) is hypothesized mainly to be a biomarker of white matter lesions [7]. Its elevated concentrations are linked to axonal damage in neurological diseases like multiple sclerosis [8] and amyotrophic lateral sclerosis [9]. Disease-modifying therapies have already been shown to lower NFL levels in multiple sclerosis which was associated with improved clinical and radiological outcome [10, 11]. Neuroimaging studies show pathological white matter changes already in premanifest and early HD [12].

Total tau (hereafter referred to as tau) is primarily an axonal protein with microtubule stabilizing function which has gained use as a biomarker in neurodegenerative diseases, most notably in Alzheimer´s disease [13]. Accumulating evidence from studies with different methodologies suggest HD is a secondary tauopathy, where neurofibrillary tangles are overrepresented in later stages as well as in a phenotype with prominent dementia (for a review see [14]). CSF studies have found elevated levels of NFL [15–17] and tau [16–19] in HD and that these are possible biomarkers for disease stage [16, 17, 19].

This study aims to compare NFL and tau, head-to-head, in an HD-cohort to validate their role as biomarkers for disease progression in HD.

## 2. Materials and methods

### 2.1 Definition of participants and clinical assessment

The participants were recruited from the HD clinic at Uppsala University Hospital and were either premanifest gene expansion carriers or manifest HD subjects. Premanifest gene expansion carriers were defined as individuals with a CAG-expansion (>35 repeats) of the HD-gene and with a diagnostic confidence level (DCL) below 4 [20]. Manifest HD subjects were defined as individuals with a CAG-expansion (>35 repeats) in the HD gene and a DCL of 4 [20].

Clinical assessment included the Unified HD Rating Scale ´99 (UHDRS) total motor score (TMS) [21], total functional capacity (TFC) [22], stroop word matching task, stroop color matching task, stroop interference, symbol-digit modality test (SDMT) [21], letter verbal fluency test [23], category fluency test (animals) [24]. Disease burden was calculated according to CAG-repeat number and age, using the formula (CAG-35.5) x age [25]. 5-year probability of disease onset was determined by age and CAG-repeat number [26]. Clinical assessment took place on the day of CSF collection by experienced clinical HD raters.

The study was conducted in accordance with the declaration of Helsinki and was approved by the regional ethical review board in Uppsala, Sweden (DNR 2012/274). All participants signed an informed consent before study entry.

### 2.2 CSF sample collection and handling

CSF was collected by lumbar puncture according to a standardized protocol at Uppsala University Hospital. The CSF was put on ice before centrifugation at 1300 G for 10 minutes at 4 degrees Celsius. The acellular proportion was stored at -70 degrees Celsius until the time of analysis. Polypropylene tubes (Sarstedt) were used throughout the procedures of collecting and storage of CSF to avoid protein adsorption. The time of day for sampling varied and there was no standard of fasting before the procedure.

### 2.3 Biochemical analyses

CSF NFL levels were measured using a sandwich ELISA method (NF-light® ELISA, Uman Diagnostics, Umeå, Sweden) as described previously in detail [27], while CSF tau levels were measured using the INNOTEST ELISA method (hTau Ag, Fujirebio Europe, Belgium) as described previously [28]. All samples were analyzed in one batch by board-certified laboratory technicians who were blinded to clinical information.

### 2.4 Statistical analyses

Tests for normality of distribution included Shapiro-Wilk in conjunction with inspection of histograms and the skewness statistic. Age was normally distributed but NFL and tau levels were not. A Mann-Whitney U test of the effect of sex category on tau and NFL concentrations was performed.

The correlations between tau and NFL versus clinical test scores, were evaluated using Spearman rank correlation coefficients. In addition to univariate correlation the Spearman correlation coefficients were adjusted for age and disease burden, respectively. Statistical significance was defined by a p-value of less than 0.05.

In order to evaluate the difference in NFL and tau between manifest and premanifest HD two separate analyses of covariance models were calculated, adjusted for age.

The p-values for the age-adjusted correlations between NFL and the independent factors were adjusted for multiplicity using the Bonferroni-Holm method, and all other reported p-values are unadjusted.

Statistical analyses were performed using R version 3.2.3. Graphs were created with GraphPad Prism version 7.

## 3. Results

The study enrolled 23 participants (mean age 42.7; standard deviation [SD] 14.4 range 19–72 years; Table 1). Of these, 11 (47.8%) were premanifest gene expansion carriers—12 (52.2%) were manifest HD subjects. The characteristics of both subgroups are described in Table 1.

**Table 1:** Characteristics of the study population.

Group	N	Age Mean (SD)	Male: female ratio	CAG Mean (SD)	Disease burden Mean (SD)	TFC Mean (SD)	TMS Mean (SD)
**Total**	23	42.7 (14.4)	13:10	43.7 (3.3)	326.3 (94.7)	11.4 (2.8)	17.3 (20.4)
**Premanifest HD**	11	33 (9.6)	6:5	44.1 (4.1)	261.2 (78.7)	13 (0)	1.3 (1.3)
**Manifest HD**	12	51.5 (12.4)	8:5	43.5 (2.6)	385.6 (65.6)	9.9 (3.2)	32.1 (18.4)

HD, Huntington’s Disease; SD, Standard deviation; CAG, CAG expansion length; TFC, Total Functional Capacity; TMS, Total Motor Score; Disease burden, (CAG-35.5) x age.

### 3.1 NFL and tau concentrations

**Fig 1:**
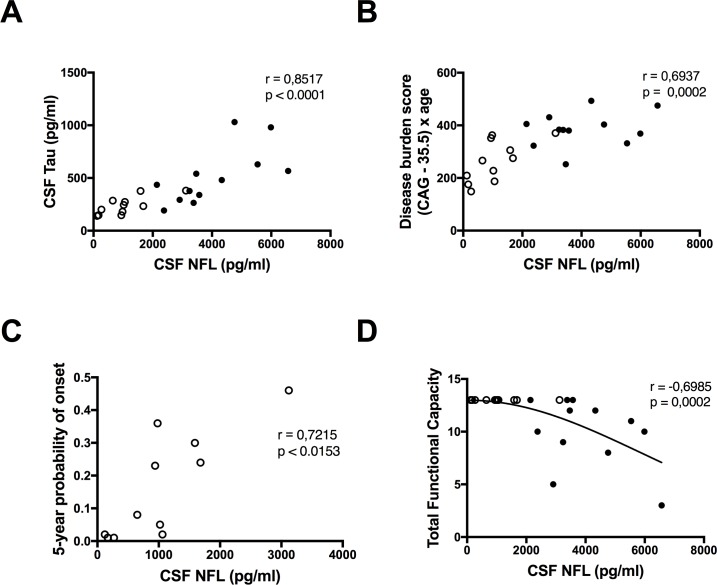
Correlations between NFL and disease progression in Huntington’s disease. (**A**) Neurofilament light (NFL) and tau levels are significantly correlated. NFL correlates positively with (**B**) disease burden and (**C**) 5-year probability of disease onset but negatively with (**D**) total functional capacity. ○ Premanifest gene expansion carrier • Manifest Huntington’s disease.

**Fig 2:**
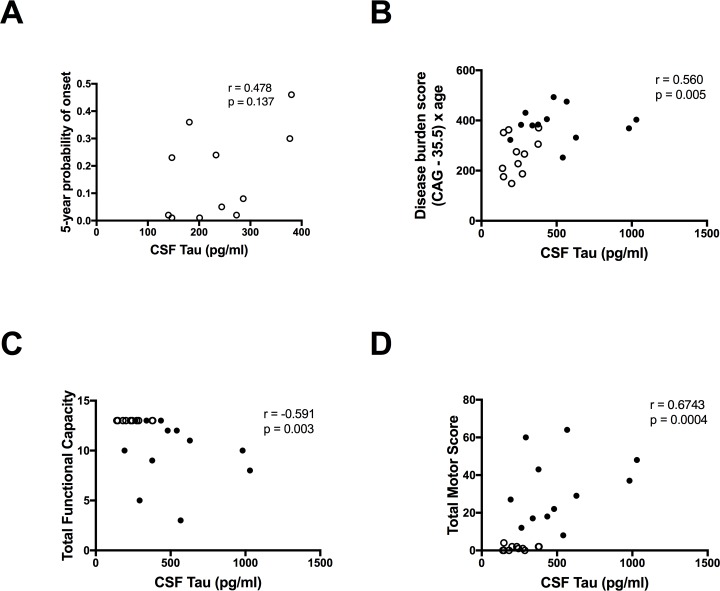
Correlations between tau and disease progression in Huntington’s disease. Tau does not correlate with (**A**) 5-year probalility of disease onset but correlations with (**B**) disease burden, (**C**) total functional capacity, and (**D**) total motor score were significant. ○ Premanifest gene expansion carrier • Manifest Huntington’s disease.

The concentrations of NFL were significantly higher in the manifest HD patients compared with the premanifest gene expansion carriers after adjustment for age (p = 0.003) but there was no significant difference between the two groups in the concentrations of tau after adjustment for age (p = 0.416). The concentrations of NFL and tau were significantly correlated (r = 0.85 p, < 0.0001) (Fig 1A). There was a significant correlation with disease burden for NFL (r = 0.69, p < 0.01) (Fig 1B) and tau (r = 0.56, p < 0.01) (Fig 2B). NFL correlated with 5-year probability of disease onset in the premanifest gene expansion carriers (n = 11 r = 0.72 p = 0.0153) (Fig 1C) but the correlation for tau was not significant (n = 11 r = 0.48, p = 0.1373) (Fig 2A).

Three young presymptomatic gene expansion carriers, who were among those farthest from predicted disease onset, had normal NFL levels according to the laboratory’s age-stratified reference range [29]. There was no association between sex and concentrations of tau or NFL (p = 0.74).

### 3.2 NFL and tau correlations with clinical testing

**Fig 3:**
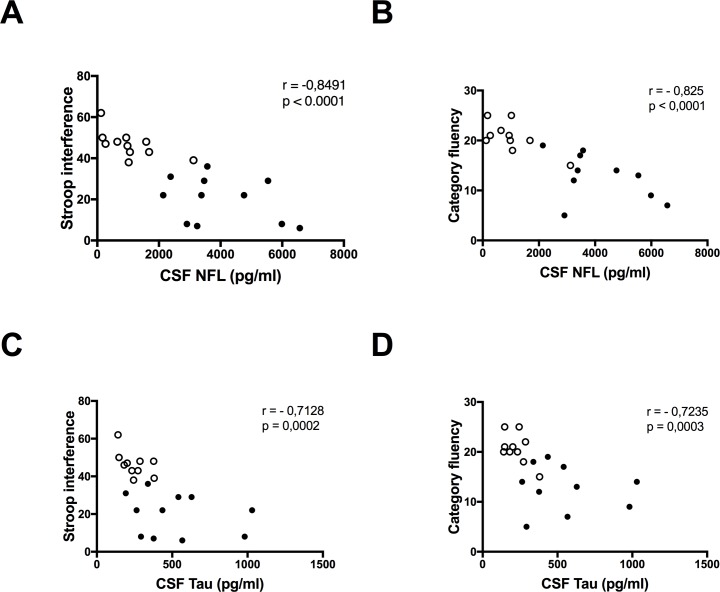
Comparing the correlations of neurofilament light and tau with cognitive test scores. Neurofilament light (NFL) has stronger correlations with clinical scores like (**A**) stroop interferens, and (**B**) category fluency, compared with (**C** and **D**) tau. ○ Premanifest gene expansion carrier • Manifest Huntington’s disease.

NFL showed strong correlations with all tests in the clinical assessment. After adjustment for age correlations for 6 out of 8 tests scores remained significant–correlations with total functional capacity (Fig 1D) and symbol digit modality were no longer significant. Correlations with total motor score, stroop color, stroop interference (Fig 3A) and letter verbal fluency remained significant after adjustment for multiplicity using the Bonferroni-Holm method. None of the NFL correlations survived adjustment for disease burden (Table 2).

**Table 2:** Spearman correlations between NFL and clinical test scores adjusted for age and adjusted for disease burden compared with the direct correlations.

	Partial Correlation (age)	p-value	Partial Correlation (disease burden)	p-value	Direct correlation	p-value
**Total motor score**	0.63	<0.01*	0.27	0.23	0.83	<0.01
**Stroop color**	-0.56	0.01*	0.02	0.93	-0.82	<0.01
**Stroop word Reading**	-0.46	0.04	0.10	0.67	-0.75	<0.01
**Stroop interference**	-0.64	<0.01*	-0.22	0.35	-0.85	<0.01
**Category fluency**	-0.56	0.01	-0.21	0.40	-0.83	<0.01
**Verbal fluency—letters**	-0.60	<0.01*	-0.27	0.25	-0.75	<0.01
**Symbol-digit modality test**	-0.34	0.14	0.11	0.65	-0.76	<0.01
**Total functional capacity**	-0.31	0.17	-0.05	0.84	-0.70	<0.01

* significant after adjustment for multiplicity using the Bonferroni-Holm method.

Moderate to strong correlations were found between tau and all the clinical scores included (Figs 2 and 3), but the magnitudes were slightly lower compared to those of NFL (Fig 3). None of the tau correlations survived adjustment for age, or disease burden score (Table 3).

**Table 3:** Spearman correlations between tau and clinical test scores adjusted for age and adjusted for disease burden compared with the direct correlations.

	Partial Correlation (age)	p-value	Partial Correlation (disease burden)	p-value	Direct correlation	p-value
**Total motor score**	0.31	0.15	0.03	0.88	0.67	<0.01
**Stroop color**	-0.27	0.24	0.12	0.60	-0.67	<0.01
**Stroop word Reading**	-0.25	0.27	0.09	0.71	-0.63	<0.01
**Stroop interference**	-0.38	0.09	-0.08	0.73	-0.71	<0.01
**Category fluency**	-0.35	0.14	-0.08	0.76	-0.72	<0.01
**Verbal fluency—Letters**	-0.29	0.20	-0.02	0.92	-0.59	<0.01
**Symbol-digit modality test**	-0.21	0.37	0.04	0.87	-0.65	<0.01
**Total functional capacity**	-0.14	0.55	0.03	0.89	-0.59	<0.01

## 4. Discussion

This study found significantly increased levels of NFL in manifest HD compared to premanifest gene expansion carriers. Tau levels had a similar tendency, although not significant after adjustment for age.

The following two sections will discuss the ability of NFL and tau to predict the clinical phenotype in HD, and compare these results with previous findings to analyze their relevance.

### 4.1 Previous findings

The first study on NFL enrolled only manifest HD patients (n = 35) and found that they had significantly higher levels of NFL in CSF compared with age and gender matched controls [15]. Out of 14 items in the clinical test battery, only the total functional capacity was significantly correlated with NFL levels after adjustment for age. Another study focused on evaluating a novel assay for quantifying mutant huntingtin (mtHTT) in CSF but also performed analyses of NFL (n = 14) and tau (n = 24) including both premanifest gene expansion carriers and manifest HD patients. Interestingly, mtHTT had a positive linear association with NFL and tau respectively (perhaps indicating that mtHTT was leaking out of degenerating neurons). NFL had correlations with cognitive test scores of similar magnitude as those of mtHTT but the correlations of NFL did not survive adjustment for disease burden, perhaps due to fewer available samples. Even though the sample size for tau was the same as for mtHTT, correlations with clinical test scores did not survive adjustment for disease burden, indicating tau may not have the same predictive ability on phenotype as mtHTT [16]. A recent study of tau included a larger sample (n = 52) and found significant correlations between tau concentrations, total functional capacity, total motor score and cognitive test scores after adjustment for age and in the majority of these tests, the correlations remained significant after adjustment for disease burden as well [19]. Recent findings in a study by Vinther-Jensen et al [17] (n = 80) support NFL as a correlate for disease burden, with levels rising already in the premanifest stage, where tau levels were only elevated in the premanifest gene expansion carriers with psychiatric symptoms compared to those without. Total motor score was correlated with NFL levels after adjustment for disease burden.

### 4.2 Neurofilament light subunit correlates stronger with disease progression compared with tau

This study strengthens the case for NFL as a biomarker of disease stage which was correlated to 6/8 items in the UHDRS assessment after adjustment for age. Total motor score, stroop color, stroop interference and letter verbal fluency remained significant after adjustment for multiplicity. The correlations with clinical scores were stronger than in the first study published that used an older ELISA with lower precision [15], and of the same magnitude as in a more recent study [16].

Our study, in agreement with two previous studies [16, 17], found NFL levels in premanifest gene expansion carriers that increase in the prodromal stage as the 5-year probability of disease onset rises, which indicates ongoing axonal damage in the white matter. This is in agreement with previous knowledge about early involvement of white matter before clinical onset of HD. However, we note that those individuals farthest from predicted clinical onset had normal NFL values (according to published reference values [29]). In agreement with a recent study [17], we found no correlation between tau and 5-year probability of disease onset, which indicates that tau is less suitable than NFL in the premanifest stage.

The magnitude of correlations between tau and disease stage were similar in our study compared to Rodrigues et al. [19], but correlations did not survive adjustment for age or disease burden.

Overall, these results suggest that, compared to tau, NFL is superior in the ability to predict the clinical phenotype in HD.

The most important limitation to this study is the small sample. Some trends suggested herein may prove significant in larger materials. Timing of meals and the time of day for sampling varied, but this is not likely to influence the results, at least regarding tau [30]. To avoid such limitations, we would like to recommend the HD Clarity project which is a new initiative for a multicenter collection of HD CSF which aims to enroll a large number of participants allowing statistical power for multiple analyses while offering a standardized protocol for CSF collection.

Disease burden is likely a confounding factor for the direct correlation between any adequate biomarker and disease stage in HD. It is therefore no surprise that adjustment for disease burden weakens the correlations of tau and NFL with clinical scores sizably. After all, the natural history of HD is governed by disease burden to a large extent [26]. Keeping in mind that disease burden is unchangeable, the correlations seen in this study are strong and suggest that especially NFL could be valuable for monitoring disease activity. While measurement of CSF mtHTT may offer proof of concept for huntingtin lowering drugs, it does not directly reflect neurodegeneration like the biomarkers in this study. Therefore, we suggest that NFL and possibly tau be used in clinical drug trials as biomarkers of disease progression that are potentially influenced by future disease-modifying therapies.

## Supporting information

S1 Table Dataset including clinical characteristics of study participants. (XLSX)
